# Poor subjective sleep reported by people living with HIV is associated with impaired working memory

**DOI:** 10.1515/nipt-2023-0010

**Published:** 2023-07-11

**Authors:** Natalie M. Zahr, Edith V. Sullivan, Adolf Pfefferbaum

**Affiliations:** Department of Psychiatry and Behavioral Sciences, Stanford University School of Medicine, Stanford, CA, USA; Neuroscience Program, SRI International, Menlo Park CA, USA; Department of Psychiatry and Behavioral Sciences, Stanford University School of Medicine, Stanford, CA, USA; Neuroscience Program, SRI International, Menlo Park CA, USA

**Keywords:** Global Assessment of Functioning (GAF), Medical Outcomes Study (MOS), Pittsburgh Sleep Quality Index (PSQI), quality of life (QoL), working memory (WM)

## Abstract

Poor sleep can undermine health and may be especially disruptive to those with chronic conditions including HIV infection. Here, clinically well-described people living with HIV [PLWH] (74 men, 35 women) and healthy control (38 men, 35 women) participants were administered the Pittsburgh Sleep Quality Index (PSQI), a validated measure of subjective sleep with a global score ≥5 able to distinguish good from poor sleepers. In addition, participants completed a battery of neuropsychological tests. PLWH (6.8 ± 3.7) had higher global PSQI scores than healthy controls (4.1 ± 2.8): 39.7 % of uninfected controls and 68.8 % of PLWH had a PSQI≥5 indicative of poor sleep. There were no relations between the global PSQI score and any evaluated variables among uninfected individuals or with demographic or HIV-related variables in PLWH. Instead, a higher global PSQI score among PLWH was associated with worse “Quality of Life” scores [Global Assessment of Functioning (GAF, p=0.0007), Medical Outcomes Study survey (21-item short form, SF-21, p*<*0.0001), and Activities of Daily Living-Instrumental (ADL-I, p=0.0041)] and higher Beck Depression Index (BDI, p*<*0.0001) depressive symptoms. Further, in PLWH, higher global PSQI scores were associated with poor performance on a working memory task, the digit backward span (p=0.0036). In PLWH, the 5 variables together explained 32.3 % of the global PSQI score variance; only 3 variables – the SF-21, BDI, and digit backward scores – explained 30.6 % of the variance. To the extent that poor subjective sleep contributes to impaired working memory in HIV, we speculate that this impairment may be ameliorated by improved sleep health.

## Background

The United States Department of Health and Human Services recognizes poor sleep among leading factors that undermine the health of persons living with HIV [PLWH] [1]. Estimates of poor sleep in the HIV community (30–73 %) exceed that in the general population (6–33 %) [2–5] and poor sleep in PLWH may be more debilitating than in others [6–8]. Polysomnography studies are the gold standard for evaluation of sleep architecture, but requirements for overnight participation and complex data analyses procedures have historically precluded sufficiently-sampled evaluations (cf., [9–11]). The Pittsburgh Sleep Quality Index (PSQI) is a well-validated measure of subjective sleep quality [12–14] that has been used in a variety of populations [e.g., 12, 15–19] with a global score ≥5 distinguishing good from poor sleepers [20]. Although subjective sleep indices such as the PSQI do not necessarily align with polysomnography measures [e.g., 21], they may nevertheless screen relevant aspects of sleep [e.g., [22, 23]].

Studies in the general population suggest potential relations between poor sleep and impaired cognitive performance [e.g., 24, 25]. Among healthy study participants, sleep restriction can induce attention and working memory lapses and depression of mood [26, 27]. A study using the PSQI in healthy young adults reported that scores ≥5 were associated with worse performance on tests of verbal learning and memory and processing speed [28]; in another study, associations between subjective sleep indices and cognitive performance were not forthcoming [29]. In PLWH, relations between poor sleep and cognition are also equivocal [cf., 22, 30–35]. In 36 PLWH (75 % men), polysomnography measures (e.g., sleep efficiency, wake after sleep onset), were associated with scores on several neuropsychological tests (e.g., Trails B, Digit Symbol Substitution, Letter Number Sequence) [36]. In a similar study of PLWH (n=32), some polysomnography measures of poor sleep (e.g., respiratory disturbance) were associated with low performance on tests of learning and memory (e.g., California Verbal Learning Test), whereas other sleep measures (e.g., sleep maintenance efficiency) were related to poor working memory (e.g., digit span backwards) [37]. Comparably, PSQI-determined poor sleep in PLWH has been associated with compromised cognitive performance on the mini mental state exam [38] and tests of learning and memory [39], but studies do not always support relations between PSQI and cognitive performance [31].

Other commonly reported correlates of poor PSQI subjective sleep in PLWH include reduced “quality of life”, as quantified using Medical Outcomes Surveys [40] and similar screens [41–44], CD4 cell count [lower: [45, 46], higher: [47–49]], hepatitis C virus (HCV) co-infection [50, 51], and clinically-diagnosed depression [31, 52] and anxiety [38, 53]. Here, confounding variables including HIV-related factors such as HCV co-infection and clinically-diagnosed major depressive (MDD) and generalized anxiety (GAD) were considered in evaluating relations between the global PSQI and neurocognitive test performance in 109 PLWH and 73 healthy controls. The hypothesis tested was that poor PSQI-determined sleep would correlate with lower performance on tests of working memory and of episodic learning and memory.

## Methods

### Study participants

These data were collected in accordance with protocols approved by the Institutional Review Boards of Stanford University and SRI International. Written informed consent was obtained from all participants in accordance with the Declaration of Helsinki. A total of 182 individuals were administered the PSQI questionnaire between February 2013 and December 2019. The characteristics of the 2 study groups including PLWH (n=109) and healthy controls (n=73) are presented in Table 1. HIV patients were referred from local outpatient or treatment centers, recruited during presentations by project staff at relevant venues, or by distribution of flyers at community events. Comparison participants were recruited from the local community by referrals and flyers. Unrelated data from many of the individuals included in the current study were published in previous reports [54–56].

### Demographics, clinical screening, and diagnoses

Age, sex, ethnicity, and education were recorded based on self-report. Handedness was measured using the *Crovitz Handedness Inventory* [57]. Socioeconomic status (SES) was derived from the *Four-FactorIndex of Social Status*, which considers education and occupation level and wherein a lower score reflects higher status [58]. Body Mass Index (BMI) was calculated from height and weight measurements made on the day of participation. All participants were screened using the Structured Clinical Interview for DSM-IV and DSM5 (SCID) [59]. Upon initial assessment, subjects were excluded if they had a significant history of medical (e.g., epilepsy, stroke, multiple sclerosis, uncontrolled diabetes, or loss of consciousness >30 min), psychiatric (i.e., schizophrenia or bipolar I disorder), or neurological (e.g., Alzheimer’s disease) disorders. Participants were excluded for recent (i.e., past 3 months) substance dependence on any drug of abuse other than alcohol. The SCID was used to diagnose Generalized Anxiety Disorder (GAD) and Major Depressive Disorder (MDD) based on established criteria [60, 61]. During the SCID, the clinical psychologist also assigned a Global Assessment of Functioning (GAF) score – a single rating scale for evaluating the overall functioning of a participant ranging from 1 for the sickest to 100 for the healthiest individuals [62] where scores above 70 indicate positive mental health and scores below 40 are found among hospitalized psychiatric patients [63]. The clinical psychologist also determined the Karnofsky score, a tool to assess general well-being with a score of 100 reflecting normal behavior with no signs of disease and 70 indicating an inability to carry on normal activities or to do active work [64].

### Questionnaires

#### Pittsburgh Sleep Quality Index [PSQI]:

The PSQI is a 19-item questionnaire that assesses sleep over the past month including quality, latency, duration, efficiency, disturbances, use of hypnotics, and daytime dysfunction (component scores are presented in Table S1). A global PSQI score – which requires completion of all test questions – greater than 5 yields a diagnostic sensitivity of 89.6 % and specificity of 86.5 % in distinguishing good from poor sleepers [20].

#### Medical Outcomes Study (MOS), 21-item, short form (SF-21):

Health status measures such as the Medical Outcomes Study (MOS) scales have been shown to be useful in HIV infected populations [65, 66]; subsets (e.g., SF-21) are sufficient and reliable in determining clinical and functional status in PLWH [67].

#### Activities of Daily Living (ADL), instrumental and physical:

ADL includes *physical* activities such as grooming, eating, and using the toilet (9 questions, highest score=18) and *instrumental* activities such as managing finances and arranging transportation (7 questions, highest score=14) [68]. A score of 0 indicates low functioning and high scores indicate independence [69].

#### The Beck Depression Inventory [BDI]:

The BDI is a 21-item, self-report rating inventory that measures characteristic attitudes and symptoms of depression [70]. Each of the 21 items corresponding to a symptom of depression is summed to give a single score with a total score of 0–13 considered minimal, 14–19 mild, 20–28 moderate, and 29–63 indicating severe depression [71]. The BDI-II takes approximately 10 min to complete [70] and demonstrates high internal consistency, with alpha coefficients of 0.86 for psychiatric populations and 0.81 for non-psychiatric populations [72].

#### Alcohol Use Disorders Identification Test [AUDIT]:

The AUDIT was developed by the World Health Organization as a self-report screening Test to identify severity of Alcohol Use Disorders and provides an overall measure of hazardous drinking [73]. Hazardous use, dependence symptoms, and harmful use are the three symptom areas covered by the 10-item scale [74]. Total scores range from 0 to 40, and higher scores represent more intense drinking [75].

### Neuropsychological tests

#### Executive functioning:

Tests of executive functioning included **Trails B** (time to connect open circles numbered from 1–13 and letters A-L alternating between numbers and letters) [76]; **Digit Symbol** (the raw number of correct digit-to-symbol substitutions accomplished in 90 sec) [77]; and the **F-A-S** phonemic fluency test (sum of unique words beginning with the letters “F”, “A” and then “S” within 1 min for each letter) [78, 79].

#### Attention and working memory:

Tests of attention and working memory included **Trails A** (time to connect open circles numbered from 1–25); Wechsler Memory Scale-revised (WMS-R) **Digit Spans** Forward and Backwards raw score totals (verbally repeating – forwards or backwards – a string of numbers spoken aloud by tester) [80]; and WMS-R **Visual Spans** Block Tapping Forward and Backward raw score totals (participant mimics tapping on a sequence of up to nine identically spatially separated blocks first produced by the examiner) [81].

#### Visual and verbal learning and memory:

Learning and memory tests included the **Rey-Osterrieth Complex Figure** (ROCF) (immediate and delayed raw scores) and the WMS-R **Logical Memory** (immediate and delay raw scores). The ROCF evaluates nonverbal memory; participants must reproduce on paper the complex geometrical shape by memory immediately after presentation and following a 30 min delay [82]. The WMS-R logical memory subtest is a standardized assessment of narrative episodic memory. The examiner reads aloud a short story, and the participant attempts to recall the story verbatim immediately after hearing the story and again following a 30 min delay [83].

### Blood assays

Serum samples were collected and analyzed by Quest Diagnostics for HIV and HCV screening with RNA quantification (viral load) and lymphocyte panel quantification (e.g., CD4 cell count) for seropositive individuals. Other relevant blood markers were also quantified to permit calculation of the Veterans Aging Cohort Study (VACS) index based on age, CD4 cell count, and HIV-1 RNA which predicts mortality and other outcomes in PLWH [84].

### Statistics

Statistical analyses were conducted in JMP^®^ Pro 16.0.0 (SAS Institute Inc., Cary, NC, 1989–2021). Data in manuscript are presented are for the global PSQI score; supplementary tables and figures provide similar results using PSQI cutoff scores. All group comparisons used Wilcoxon χ^2^ for nonparametric evaluation. Within group correlations used χ^2^ for nominal (i.e., categorical) variables and Spearman’s ρ for continuous variables. A Bonferroni corrected value of p=0.006 was required for significant correlations (i.e., 10 HIV-related variables, i.e., p=0.05/8). Distinguishing variables were evaluated for their contribution to the global PSQI score using Akaike Information Criterion (AIC), forward stepwise regressions. The variance explaining PSQI global scores after including relevant covariates was derived from standard least squares models.

## Results

### Group differences

**Figure 1:**
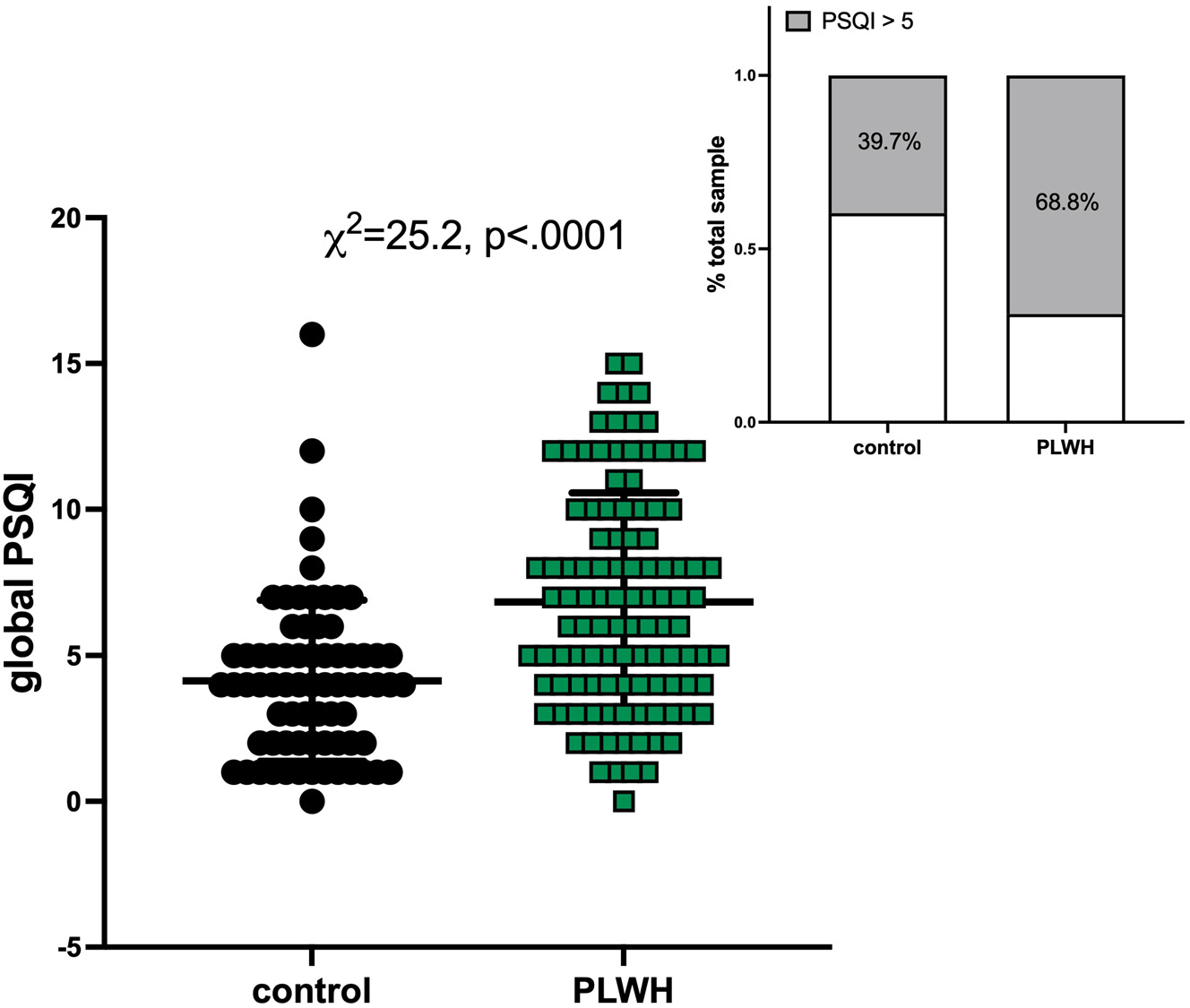
Global Pittsburgh Sleep Quality Index (PSQI) scores in healthy controls and in people living with HIV infection (PLWH). Inset demonstrates percent of total participants in each group with PSQI≥5.

PLWH relative to the control group had a greater number of African Americans, fewer years of education, and lower SES; they also had a high incidence of HCV and were more likely to smoke and drink alcohol (all p*<*0.0001, Table 1). Further, compared with the control group, PLWH were more like to be diagnosed with GAD or MDD, and had lower GAF and a higher VACS index (all p*<*0.0001, Table 1). PLWH had significantly higher global PSQI scores (6.8 ± 3.7, χ^2^=25.2, p*<*0.0001) and higher frequency of poor sleep (PSQI≥5, 68.8 %) than healthy control individuals (4.1 ± 2.8, PSQI≥5, 39.7 %) (Figure 1). Regarding PSQI component scores, PLWH relative to healthy controls had poorer overall subjective sleep quality (component 1, p*<*0.0001), significantly longer sleep latency (component 2, p=0.0002), and more daytime dysfunction (component 7, p*<*0.0001) (Table S1). Finally, PLWH relative to healthy controls had worse scores on all administered questionnaires and lower performance on all administered cognitive tests (all p*<*0.0001, except AUDIT p=0.0247 and trails A p=0.0003, Table S2).

### Correlations based on global PSQI score

**Figure 2:**
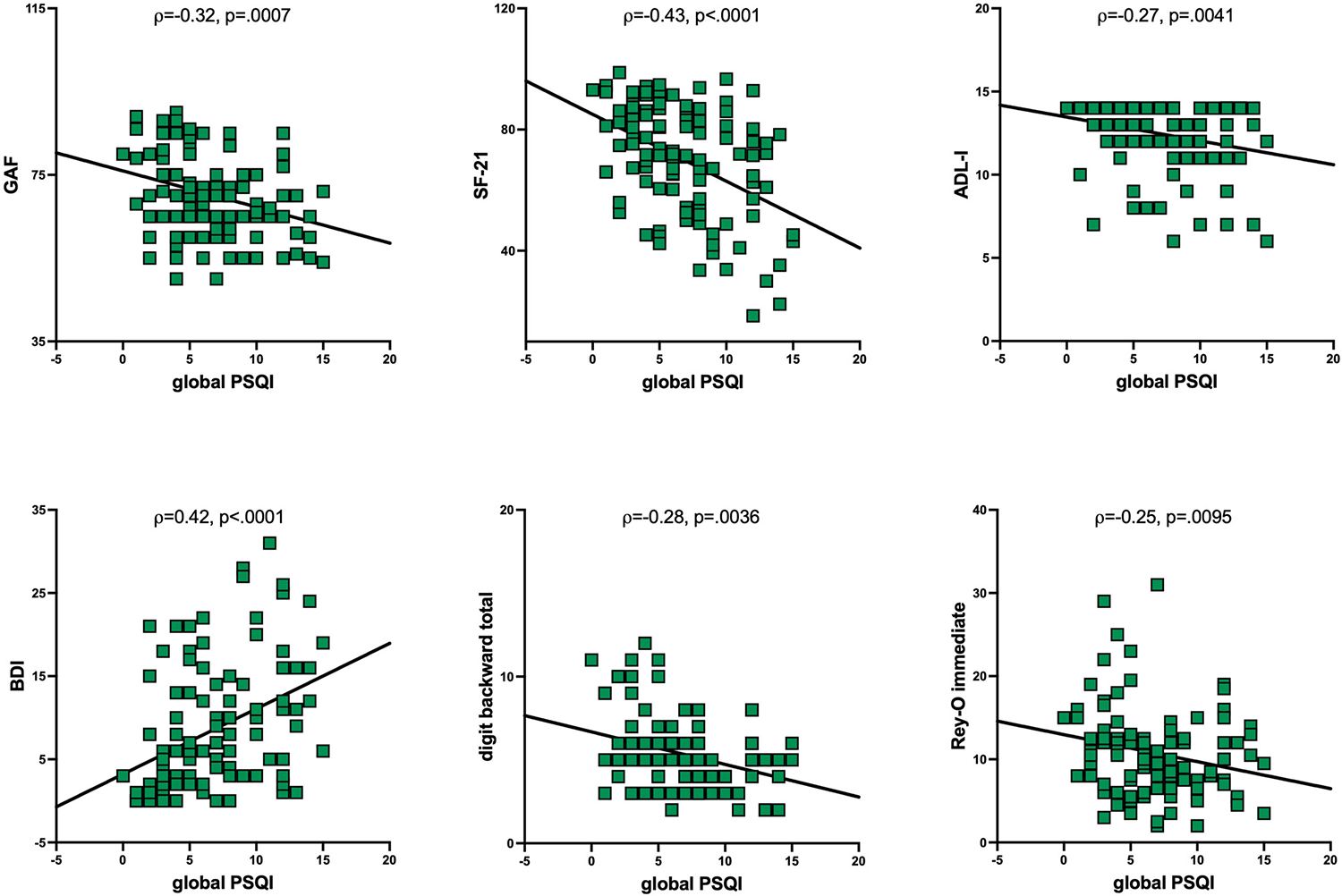
Correlates among PLWH of the global Pittsburgh Sleep Quality Index (PSQI) score including those that did not meet Bonferroni-corrected significance (p*<*0.005). ADL-I, Activities of Daily Living – Instrumental; BDI, Beck Depression Index, GAF, Global Assessment of Functioning, SF-21, medical outcomes study (MOS), 21-item, short form. (SES, socioeconomic status not pictured).

The control group did not show relations between the global PSQI score and demographic, HIV-related, clinical, or cognitive performance variables that reached Bonferroni-corrected significance (Table 2). Among PLWH, global PSQI correlates included the GAF (ρ=−0.32, p=0.0007), SF-21 (ρ=−0.43, p*<*0.0001), ADL-I (ρ=−0.27, p=0.0041), BDI (ρ=0.42, p*<*0.0001), and WMS-R digit backwards total (ρ=−0.28, p=0.0036); SES (ρ=0.25, p=0.0091) and the Rey-O immediate raw score (ρ=−0.25, p=0.0095) were also significantly associated with the global PSQI score but at p-values below the Bonferroni correction (Table 2, Figure 2). Within the PLWH group, multiple regression revealed that the 7 variables together explained 33.1 % of the variance in the global PSQI score (F_7,109_=6.8, p*<*0.0001). Only 3 of the variables isolated by a stepwise regression – the SF-21, BDI, and digits backwards – together explained 30.6 % of the variance suggesting that the remaining variables contributed only negligibly to the global PSQI score.

### Variable relations using PSQI cutoff scores

Healthy controls with PSQI≥5 (n=29) and those with better sleep (n=44) showed no group differences (Table S3, Figure S1). By contrast PLWH with PSQI≥5 (n=75) relative to PLWH with better sleep (n=34) had worse scores on the SF-21 (χ^2^=13.6, p=0.0002), BDI (χ^2^=15.8, p*<*0.0001), digits backwards total (χ^2^=7.7, p=0.0054), and Rey-O immediate raw (χ^2 2^=9.9, p=0.0017). Scores on the GAF (χ^2^=7.2, p=0.0072) and ADL-I (χ^2^=7.0, p=0.0083) were worse in PLWH with PSQI≥5 relative to those with scores below cutoffs, but these relations did not reach Bonferroni-corrected significance (Table S3, Figure S1). Within the PLWH group, a nominal logistic regression including all 6 variables explained 25.4 % of the variance in the PSQI cutoff scores (χ^2^=32.6, p*<*0.0001), in a model driven by digit backwards performance (p=0.04). Just 3 variables isolated by a stepwise regression – SF-21, BDI, and digits backwards – explained 22.5 % of the PSQI score treated as a cutoff.

## Discussion

The study reported here in 109 PLWH and 73 healthy controls supports poor PSQI-determined sleep quality in PLWH and extends the literature by demonstrating a salient relation between PSQI and digit backwards performance even after considering relevant variables. Converging results from the PSQI score treated as a continuous variable (i.e., global PSQI score) and as a nominal variable (i.e., PSQI≥5), even after accounting for statistically significant correlates reported in the literature as relevant to PSQI scores in PLWH suggest an effect of poor subjective sleep on working memory in PLWH. Working memory may be among the cognitive functions particularly affected by poor sleep [cf., 85–88]. Poor PSQI-sleep has been associated with low working memory performance in the general population [89, 90], in nightshift workers [91], in resident physicians self-reporting memory impairments [92], in bipolar disorder [93], and in schizophrenia [94].

Another significant finding from the current study is the prominent relationship between poor self-reported sleep and worse quality of life in PLWH, comporting with findings frequently reported in the literature [31, 40–43, 52, 95, 96]. Here, “quality of life” was assessed using multiple tools including the SF-21, ADL, GAF, and the Karnofsky scores [cf., 43]. While the SF-21 and ADL are based on subjective responses to questionnaires (i.e., self-report), the GAF and the Karnofsky are based on a scores assigned by an external, objective observer. Correlations between the PSQI and worse quality of life as measured by the SF-21 and the GAF suggest that both self-reported and externally perceived quality of life are impacted by poor PSQI-defined sleep.

Poor sleep as determined by the PSQI is also often reported in those with MDD [97–100], but this relation was not forthcoming here. Instead, higher global PSQI scores correlated with higher scores on the BDI, a questionnaire that evaluates depressive symptoms. In several populations, including nurses [101], those with chronic kidney disease [102], alcohol use disorder [98], and multiple sclerosis [103], correlations between PSQI and BDI scores have been reported suggesting that poor sleep can contribute to depressive symptoms. Other reports citing poor PSQI sleep correlates with CD4 count [45–49] or psychiatric diagnoses [e.g., 31, 38, 52, 53] were not replicated.

This report also revealed a significant PSQI relation with performance on the Rey-O immediate raw (recall) score, particularly when evaluated using the categorical PSQI≥5 score (note, relative to digits backwards, the Rey-O score contributed less to explaining PSQI variance). This finding comports with papers suggesting high PSQI scores are associated with poor short-term memory [e.g., 90, 104, 105].

Limitations of the current study include potential bias in the selection of participants contributing to a lack of generalizability of the enrolled sample. Another limitation is a recognized issue with the PSQI as scores do not necessarily correlate with objective sleep measures [22, 106–109]. Nevertheless, the current findings extend the literature by replicating the often-described relationship between poor sleep identified subjectively and compromised quality of life in PLWH and extends the literature by demonstrating a relationship between poor subjective sleep and worse working memory performance after accounting for relevant variables. Indeed, the current results suggest that among all PLWH, sleep is a modifiable disease target that may improve quality of life [110]. Further, assessing and treating sleep complaints in HIV might improve working memory functioning.

## Supplementary Material

supplementary material

## Figures and Tables

**Table 1:** Genaral characteristics of the 2 groups.

	Control (n= 73)	PLWH (n= 109)	Wilcoxon χ^2^	p-Value

Demographics				

Age, years	59.1 ± 10.5	57.2 ± 7.1	1.74	0.1871
Sex (men/women)	38/35	74/35	4.63	0.0314
Self-defined ethnicity^a^	44/8/21	36/55/17	30.66	**<0.0001**
Handedness (right/left)	67/6	96/13	0.62	0.7342
Body mass index (BMI)	25.4 ± 4.3	26.6 ± 4.8	3.68	0.0552
Education, years	16.7 ± 2.1	13.6 ± 2.4	57.53	**<0.0001**
Socioeconomic status (SES)^b^	21.4 ± 9.3	39.6 ± 14.2	66.75	**<0.0001**

**HIV-related variables**				

HIV onset age, years	-	34.6 ± 8.8	-	-
HIV duration, years	-	21.5 ± 7.7	-	-
Viral load (log copies/mL)	-	1.8 ± 1.0	-	-
CD4 cell count current (100/mm^3^)	-	661.0 ± 306.7	-	-
CD4 cell count nadir (100/mm^3^)	-	187.3 ± 174.7	-	-
AIDS-defining event (yes/no)	-	11/98	-	-
ART^c^ (yes/no)	-	103/6	-	-
Hepatitis C Virus (yes/no)	0/73	34/75	24.3	**<0.0001**

**Clinical characteristics**				

Smoker (yes^d^/no)	4/69	58/51	44.71	**<0.0001**
Lifetime alcohol consumption, kg	51.7 ± 78.1	551.6 ± 828.1	49.10	**<0.0001**
Generalized anxiety disorder (yes/no)	0/73	21/88	12.53	**<0.0001**
Major Depressive Disorder (yes/no)	0/73	35/74	22.87	**<0.0001**
Global Assessment of Functioning	85.3 ± 7.0	70.0 ± 10.0	79.21	**<0.0001**
Karnofsky score	100.00	98.8 ± 4.0	4.34	0.0373
VACS^e^ index	18.5 ± 13.4	33.0 ± 17.1	22.84	**<0.0001**

a Caucasian/African American/other = native American, Asian, Islander;

b lower score = higher status;

c ART = active retroviral therapy;

d past or current;

e VACS = Veterans Aging Cohort; bold = significant.

**Table 2:** Correlates of the global PSQI score within each group.

	control		PLWH	
	𝛘^2^ or 𝛒 ^a^	p-Value	𝛘^2^ or 𝛒 ^a^	p-Value

Demographics				

Age, years	−0.05	0.6797	0.03	0.7498
Sex (men/women)	0.88	0.3480	4.66	0.0308
Self-defined ethnicity	1.55	0.4614	2.89	0.2359
Handedness (right/left)	1.60	0.4485	0.12	0.9402
Body mass index (BMI)	0.20	0.0918	0.02	0.8051
Education, years	−0.17	0.1564	−0.23	0.0170
Socioeconomic status (SES)	0.30	***0.0097***	0.25	***0.0091***

**HIV-related variables**				

HIV onset age, years	-	-	0.05	0.6000
HIV duration, years	-	-	−0.08	0.4016
Viral load (log copies/mL)	-	-	−0.08	0.4627
CD4 cell count current (100/mm^3^)	-	-	0.14	0.1807
CD4 cell count nadir (100/mm^3^)	-	-	−0.11	0.3013
AIDS-defining event (yes/no)	-	-	6.67	0.1542
ART (yes/no)	-	-	0.13	0.7187
Hepatitis C virus (yes/no)	-	-	6.17	0.0130

**Clinical characteristics**				

Smoker (yes/no)	1.00	0.6056	1.64	0.4406
Lifetime alcohol consumption, kg	−0.11	0.3607	0.06	0.5689
Generalized anxiety disorder (yes/no)	-	-	6.12	0.0129
Major Depressive Disorder (yes/no)	-	-	1.53	0.2166
Global Assessment of Functioning	−0.16	0.1955	−0.32	**0.0007**
Karnofsky score	-	-	−0.14	0.1448
VACS index	−0.07	0.6387	−0.18	0.1253

**Questionaiires**				

SF-21	−0.16	0.1845	−0.43	**<0.0001**
ADL-I	0.18	0.1221	−0.27	**0.0041**
ADL-P	0.02	0.8691	−0.22	0.0240
BDI	0.16	0.1675	0.42	**<0.0001**
AUDIT	−0.08	0.5308	−0.02	0.8486

**Executive functioning**				

Trails B	0.11	0.3481	0.21	0.0350
Digit symbol (90 sec, raw)	−0.08	0.4918	−0.14	0.1599
Phonemic fluency	−0.03	0.8012	−0.15	0.1271

**Attention and working memory**				

Trails A	−0.05	0.6673	0.08	0.4090
Digit forward	−0.12	0.3044	−0.20	0.0386
Digit backward	−0.23	0.0513	−0.28	**0.0036**
Blocks forward	−0.20	0.1039	−0.06	0.5604
Blocks backward	−0.05	0.6716	−0.02	0.8625

**Verbal and visual learning and memory**				

Rey-O immediate raw score	0.12	0.3402	−0.25	***0.0095***
Rey-O delay raw score	0.13	0.2996	−0.18	0.0731
Logical memory immediate total	−0.08	0.4999	−0.10	0.3368
Logical memory delay total	−0.11	0.3676	−0.14	0.1609

a Nonparametric *x*2 Wilcoxon test for nominal variables, nonparametric Spearman’s ρ test for continious variables; bold = significant; bold italic = significant above Bonferroni correction.
