# Supplementary material for: Poor subjective sleep reported by people living with HIV is associated with impaired working memory
Source: NeuroImmune Pharm Ther. Author manuscript; Available in PMC 2023 Nov 9. (PMC10635409; doi:10.1515/nipt-2023-0010)
Supplement: supplementary material [file NIHMS1939714-supplement-supplementary_material.docx]

**Supplementary Materials**

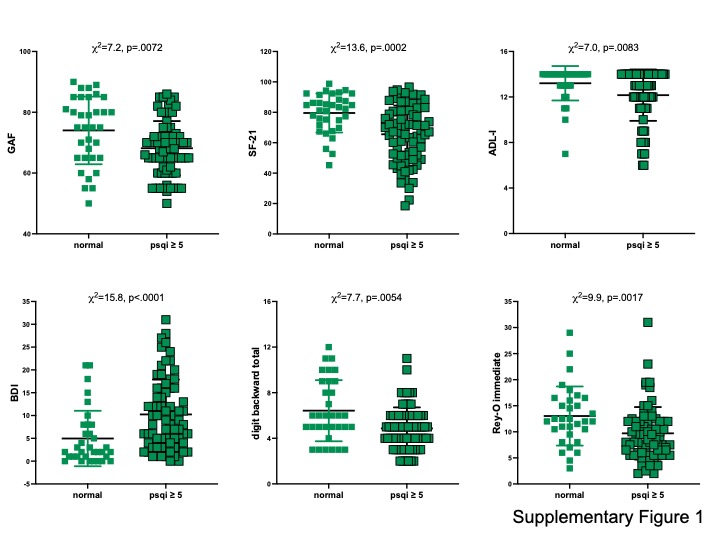


**Figure S1.** Variables different between PLWH with PSQI>5 relative to PLWH with scores below cutoff. GAF = Global Assessment of Functioning, SF-21 = Medical Outcomes Study (MOS), 21-item, short form, ADL-I = Activities of Daily Living – Instrumental, BDI = Beck Depression Index.
